# Recurrent gene conversions and subcluster-specific variants are associated with *Mycobacterium tuberculosis* adaptation to intensive host pressure: a comparative genomic study

**DOI:** 10.3389/fmicb.2026.1950486

**Published:** 2026-10-07

**Authors:** Tianyu Lin, Kun Chen, Zhenchun Chen, Yurou Jin, Weizhen Chen

**Affiliations:** 1 Department of Infectious Diseases, Fuzong Clinical Medical College of Fujian Medical University, Fuzhou, Fujian, China; 2 Department of Infectious Diseases, 900th Hospital of PLA Joint Logistic Support Force, Fuzhou, Fujian, China; 3 Department of Clinical Laboratory Medicine, 900th Hospital of PLA Joint Logistic Support Force, Fuzhou, Fujian, China; 4 Medical Information Data Room, 900th Hospital of PLA Joint Logistic Support Force, Fuzhou, Fujian, China

**Keywords:** gene conversion, structural variation, microevolution, *Mycobacterium tuberculosis*, outbreak, PE/PPE, SNP

## Abstract

**Background:**

Long-read sequencing has revealed complex variations, such as gene conversions in immunologically critical PE/PPE gene families, yet their role in *Mycobacterium tuberculosis* host adaptation remains unclear.

**Methods:**

We performed a comparative genomic analysis across a gradient of host-imposed selective pressure: chronic within-host infection, latent household transmission, and a 21-year clonal outbreak in Bern, Switzerland.

**Results:**

We observed a constrained accumulation of mutational events across distinct epidemiological settings, with rates ranging from 0.36 to 0.81 events per genome per year. Under minimal host-imposed selection, the pathogen accumulated only small-scale variants (i.e., SNPs and indels). During the outbreak, however, the genetic diversity landscape shifted toward larger-scale variants, including recurrent gene conversions and structural variations. Notably, an early PPE19-to-PPE18 conversion emerged in the ancestral strain and was maintained across the entire lineage. In contrast, 86% of variants were transient singletons, with only a small proportion persisting under selection.

**Conclusion:**

These findings suggest a framework in which *M. tuberculosis* maintains genomic stability through purifying selection, while retaining the capacity to generate adaptive diversity through large-scale genetic rearrangements in immunologically relevant genomic features under intensified host pressure.

## 1 Introduction

Tuberculosis remains a major global health burden. Whole genome sequencing (WGS) has been widely adopted for variant detection in *Mycobacterium tuberculosis* (Meehan et al., 2019). However, its resolution is inherently limited by technical constraints (Marin et al., 2022). Encouragingly, a substantial portion of previously excluded genomic positions—including 34.5% of PE/PPE family members—can be reliably called using short-read sequencing. And this finding has been corroborated by subsequent work (Gan et al., 2025). Nevertheless, even with bioinformatic strategies such as adjusting mapping quality thresholds or masking refined low-confidence (RLC) regions, a considerable fraction of the genome remains inaccessible to short-read sequencing (Marin et al., 2022, Modlin et al., 2021). Long-read sequencing platforms have significantly improved genome assembly continuity by spanning complex repetitive and structurally variable regions (Espinosa et al., 2024). Nevertheless, the substantial resource requirements currently limit its large-scale application.

In *M. tuberculosis*, the PE/PPE gene family constitutes approximately 10% of the coding genome. Emerging evidences showed that the PE/PPE family is involved in host-pathogen interaction and immune evasion (Zhang et al., 2025). However, these genes exhibit extensive sequence redundancy and highly repetitive architectures, making them largely inaccessible to short-read sequencing and typically excluded from WGS analysis (Gomez-Gonzalez et al., 2023, D'souza et al., 2025, Dahiya et al., 2025). To overcome this obstacle, several large-scale studies have explored the complete genome sequences using long-read sequencing (Behruznia et al., 2025, Espinoza et al., 2025). Nevertheless, the extensive genetic diversity within these study cohorts has made it difficult to derive detailed insights into specific evolutionary dynamics. Given this limitation, within-host microevolution has been extensively investigated using WGS (Ley et al., 2019). Recently, long-read sequencing has been applied to examine microevolution in latent infections (Osugi et al., 2024). Most notably, a study of a clonal outbreak demonstrated that transient singletons arising from PE/PPE gene conversion are a major contributor to genetic diversity (Stritt et al., 2025). Under current analytical frameworks, however, automated annotation of complex repetitive loci remains a bioinformatic challenge. Therefore, the full landscape of genetic variation has remained elusive, and the biological significance of these singletons is still unclear.

Building upon this work, we first reconstructed the phylogeny of the clonal outbreak strains. Then we performed systematic pairwise comparisons against the ancestral genome, to comprehensively delineate genomic variation. This analysis identified recurrent PE/PPE gene conversions and subcluster-specific SNPs. To place these observations in a broader epidemiological framework, we extended our analysis to include chronic within-host evolution and latent household transmission.

## 2 Methods

### 2.1 Study design and data sources

This study used long-read sequencing data to analyze *Mycobacterium tuberculosis* microevolution across a gradient of host-imposed selective pressure: chronic within-host infection, latent household transmission, and a prolonged clonal outbreak. Ethical approval was obtained from the Institutional Review Board of the 900th Hospital of PLA Joint Logistic Support Force (Approval No. 2025–183), and written informed consent was obtained from the participant who provided samples.

- Within-host evolution cohort: Three *M. tuberculosis* strains were sequentially isolated from sputum (June 2019, June 2021, March 2022) of a male patient with pulmonary tuberculosis. The patient had poorly controlled diabetes (HbA1c 9·3–10·8%) and exhibited poor adherence to anti-tuberculosis therapy.
- Household transmission cohort: This cohort comprised publicly available sequencing data from a study of latent household transmission in Japan (Osugi et al., 2024).
- Clonal outbreak cohort: Public data from an *M.tuberculosis* outbreak in Bern, Switzerland, were included (Stritt et al., 2025, Stucki et al., 2015). These data comprised high-fidelity long-read (PacBio) data for 16 isolates and Illumina short-read data for 74 strains, with substantial isolate overlap between the two datasets.

### 2.2 *M. tuberculosis* culture, genome sequencing and assembly

For the novel within-host strains, frozen stocks of *M. tuberculosis* strains were revived by inoculation onto Löwenstein-Jensen (L-J) slants and incubated at 37 °C under a 5% CO₂ atmosphere, which provides optimal growth conditions for *M. tuberculosis* (Caulfield and Wengenack, 2016). From the cultured colonies, a single colony was randomly selected and inoculated into Middlebrook 7H9 liquid medium using the BACTEC MGIT 960 system, which maintains the same incubation conditions. Although this approach may capture only a subset of the within-host population diversity, it was nevertheless employed (Black et al., 2015).

Short-read (2 × 150 bp) and long-read (PacBio HiFi) libraries were constructed and sequenced on Illumina NovaSeq 6,000 and PacBio Sequel II platforms, respectively. PacBio HiFi reads were assembled *de novo* using Flye (v2.9.5) (Kolmogorov et al., 2019), and the resulting contigs were polished with the Illumina reads using Pilon (v1.24) (Walker et al., 2014). Given the high sequencing depth (>1,400 x) for the three sequential strains, the longest subset of reads representing 500 x coverage was used as seeds for assembly (parameter: --asm-coverage 500). Raw data for the latent household transmission cases were processed through equivalent pipelines.

For the Bern outbreak cohort, the complete genome assemblies generated in the original long-read study were directly utilized. In the original study, assembly validation and manual curation were performed by aligning long reads back against the respective *de novo* assemblies and correcting identified inconsistencies. To verify the accuracy of the identified variants, we downloaded the PacBio HiFi raw data and aligned the long reads back against the assembly of ancestral strain 3 using minimap2 (version 2.30-r1287) (Li, 2018). Read-assembly consistencies were then scrutinized using the Integrative Genomics Viewer (IGV, version 2.11.9) (Robinson et al., 2011). Consistent with expectations, all reported variants were confirmed in this manner. Of note, a number of variants were observed at heterogeneous frequencies. Illumina short reads from the remaining outbreak strains were independently mapped to the H37Rv reference genome (NC_000962.3). To standardize genome orientation, all complete assemblies were reoriented to start from the dnaA gene using the fixstart tool in Circlator (version 1.5.5) (Hunt et al., 2015).

### 2.3 Comparative genomics and variant characterisation

Strain lineage assignment followed the hierarchical SNP-based typing system described by Shitikov and Bespiatykh (2023). Strains classified as modern Lineage 2 were further subtyped using a complementary scheme (Zhu et al., 2023). We identified newly acquired variants in derived strains by comparing them to the ancestral strain. Variants were then annotated against the H37Rv reference genome. The workflow proceeded as follows: (i) All variants in each derived strain relative to the ancestral strain were identified via pairwise alignment using NucDiff (version 2.0.3) (Khelik et al., 2017), which reports coordinates in both query and reference genomes. (ii) The same derived strains were also aligned pairwise against H37Rv. To assign H37Rv coordinates to derived-specific variants, the derived strain’s genome served as a bridging reference. Variants common to both comparisons, as determined by identical coordinates in the derived genome, were annotated using SnpEff (version 5.2) (Cingolani et al., 2012) with respect to H37Rv. Unmatched variants and all structural variants underwent manual curation. The variants in *grcC1* (Stritt et al., 2025) and *glpK* (Vargas et al., 2021), implicated in adaptive phenotypes *in vitro*, were excluded from analysis. Identified variants were classified by their molecular mechanisms.

### 2.4 Calculation of mutation event rates

We used long-read derived genomic data to estimate mutation event rates across different epidemiological settings. Briefly, a mutational event was defined as any independently occurring genetic alteration, and each event was counted once regardless of the number of nucleotide sites affected. For each isolate or transmission pair, the rate was calculated as the number of events divided by the time interval between the available isolation dates, expressed as events per genome per year. Statistical comparisons were performed using Mann–Whitney U tests in SPSS (version 22), with a two-tailed *p*-value <0.05 considered statistically significant. A detailed description of the workflow is provided in Supplementary File S1.

### 2.5 Short-read based phylogenetics and SNP mining for the outbreak

Short-read data for all 74 Bern outbreak strains were processed using an established pipeline with minor modifications (Vargas et al., 2023): (i) Raw reads were quality-trimmed using Sickle. (ii) Trimmed reads were aligned to the H37Rv reference using BWA-MEM2 (version 2.2.1) (Vasimuddin Md et al., 2019). (iii) SAM files were sorted, converted to BAM, and deduplicated using Picard (version 2.27.5). (iv) BAM files were indexed with Samtools (version 1.10) (Danecek et al., 2021) and processed with Pilon (version 1.24) (Walker et al., 2014) (parameters: --variant --minmq 40) to generate VCF files.

Only variants meeting all of the following criteria were retained: (i) labeled as PASS by Pilon; (ii) mean base quality (INFO.BQ in VCF) > 20; (iii) no supporting INDEL evidence at the locus; (iv) read depth (INFO.DP) ≥ 20; (v) proportion of reads supporting a single allele (INFO.QP) > 75%. Additionally, SNPs located within refined low-confidence (RLC) regions were excluded (Marin et al., 2022). For phylogenetic inference, high-quality SNPs were concatenated. Multiple sequence alignment was performed with MAFFT (version 7.453) (Katoh and Standley, 2013), and a maximum-likelihood tree was constructed using IQ-TREE (version 1.6.12) (Nguyen et al., 2015). Phylogenetic trees were visualized with iTOL^1^ (Letunic and Bork, 2024).

## 3 Results

### 3.1 Negligible genomic variation during within-host microevolution

To investigate the intrapatient microevolution under minimal antibiotic pressure, we analyzed three sequentially isolated strains from a patient with chronic pulmonary tuberculosis and poor treatment adherence. Each assembly was approximately 4.4 Mb in length and displayed a uniform GC content of 65.61% (Supplementary Table S1). Phylogenetic classification assigned all three strains to lineage L2, sublineage M6.2 (Shitikov and Bespiatykh, 2023), which also corresponds to the L2.3.1 sublineage (Zhu et al., 2023).

Pairwise genomic comparisons revealed only rare genetic divergence. Notably, a 5,968 bp genomic segment present in strain 211,397 was absent in both 191,350 and 227,375 (Table 1). Given that this fragment is conserved in reference strains H37Rv and CDC1551 (AE000516.2), its presence likely represents the ancestral state.

**Table 1:** Genetic variants in serial *M. tuberculosis* isolates.

Position†	Gene	191,350	211,397	227,375	Annotation
166,934	Rv0140	WT	C > T	WT	Missense
3,975,932	kstD	WT	C > T	WT	Synonymous
2,234,969–2,240,936	--	5,968 bp del	WT‡	5,968 bp del	Deletion
820,842	serA2	WT	WT	GTGA > G	Disruptive inframe deletion

† Genomic positions are reported relative to the *M. tuberculosis* H37Rv reference genome (NC_000962.3). ‡ Strain 211,397 likely represents a paralogous subpopulation, as it retains a 5,968 bp genomic segment that is conserved in reference strains H37Rv and CDC1551 but absent in the other two isolates.

The retention of this region suggests that 211,397 may have originated from a paralogous subpopulation within the same host, consistent with previous reports demonstrating that *M. tuberculosis* can diversify into coexisting sublineages within a single patient (Lieberman et al., 2016). This isolate was therefore excluded from the mutation event rate analysis. The comparison between strains 191,350 and 227,375 uncovered only a single GTGA > G deletion, yielding a mutation rate of 0.36 events per genome per year (Tables 1, 2). Given the remarkably limited genomic divergence across all three isolates, the observed clinical deterioration is more plausibly attributed to diabetes-exacerbated host immune impairment than to pathogen-driven microevolution.

**Table 2:** Molecular mechanisms of genetic variation in *M. tuberculosis* across different epidemiological contexts.

Pair/strain	Years	Snps	Indels	Homopolymeric tracts	Complex mutations	Gene conversions	Gene conversions (variants) *	Structure variants
Within-host pair								
191,350/227,375	2.8		1					
Latent household pairs								
pair 1	5.6	3						
pair 2	6.2	4						
pair 3	6.8	3				1	2	
pair 4	6.6	5	1					
pair 5	5.5	7						
pair 7	3.9	2	2					
Clonal outbreak strains								
strain 5	3	3						1
strain 7	4	1						
strain 12	7	2		1	1	1	3	
strain 8 †	NA	1						
strain 11	8	1						4
strain 1	3	3		1		1	63	2
strain 9 †	NA	1						
strain 15	10	2		1				1
strain 2	3	3	1	1		1	6	
strain 4	17	6						
strain 6	3	1	1					
strain 10 ‡	7	5						1
strain 13	14	1				2	32	1
strain 14	15	4						

* Number of nucleotide variants introduced by each gene conversion.

† Strains 8 and 9 were excluded from mean value calculations due to unclear temporal context.

‡ Strain 10 represents a paralogous lineage and was excluded from downstream analyses.

### 3.2 Limited genomic divergence during latent household transmission of *M. tuberculosis*

To explore interpatient microevolution during latent infection, we analyzed genomic sequences from seven household transmission pairs in Japan. In each pair, the secondary case developed active tuberculosis more than 3 years after the index case, consistent with reactivation following prolonged latency (Supplementary Table S2). One isolate, JPN-OP-6-1 in pair 6, exhibited substantially reduced short-read coverage (97.85%) across a 758,813 bp contig in its long-read assembly, raising concerns about potential incompleteness. Consequently, this pair was excluded from analyses. Phylogenetic analysis assigned the majority of strain pairs to the ancient L2 sublineage, specifically clades L2.2.AA1, L2.2.AA2, and L2.2.AA3, which are known to predominate in Japan (Table 3; Iwamoto et al., 2023). In pair 4, three SNPs were detected as low-frequency variants in the index case, with allele frequencies ranging from 25 to 27% (Table 3). These variants were transmitted to the secondary case and subsequently fixed, consistent with previous reports (Seraphin et al., 2019, Walter et al., 2025, de Zwaan et al., 2025). As they did not represent adaptive mutations acquired during the secondary infection, these variants were excluded from downstream analyses.

**Table 3:** Genetic variants identified in paired *M. tuberculosis* isolates from latent household infections.

Pair	Interval (yr)	Strains	SMRT	Illumina	Lineage	Variant type	Position *	Gene	REF†	ALT	Annotation
1	5.6	JPN-OP-1-1	SRR26139274	SRR26139268	L2.2. AA3.1	SNP, back-mutation	2,549,699	Rv2277c	*C*	*T*	Missense
		JPN-OP-1-2	SRR26139273	SRR26139267	L2.2. AA3.1	SNP	2,498,912	Rv2226	G	A	Synonymous
						SNP	3,188,151	Rv2877c	C	T	Missense
2	6.2	JPN-OP-2-1	SRR26139262	SRR26139266	L2.2. AA3.2	SNP, back-mutation	3,501,132	-	*T*	*C*	Intergenic
		JPN-OP-2-2	SRR26139253	SRR26139265	L2.2. AA3.2	SNP	1,183,630	Rv1060	A	G	Synonymous
						SNP	3,651,923	ctpC	A	G	Synonymous
						SNP	1,988,414	Rv1756c	C	A	Missense
3	6.8	JPN-OP-3-1	SRR26139252	SRR26139264	L4.9.1	-	-	-	-	-	-
		JPN-OP-3-2	SRR26139251	SRR26139263	L4.9.1	SNP	3,226,172	ffh	C	T	Synonymous
						SNP	265,007	Rv0221	C	G	Missense
						Gene Conversion	370,750–370,764	ppe5	-	-	PPE5 partially replaced by ppe21
						SNP	576,280	mshA	A	G	Synonymous
4	6.6	JPN-OP-4-1	SRR26139250	SRR26139261	L2.2. AA1 (Bmyc3)	SNP, back-mutation	3,741,711	pe_pgrs50	*A*	*G*	Missense
		JPN-OP-4-2	SRR26139249	SRR26139260	L2.2. AA1 (Bmyc3)	SNP	797,734	Rv0697	C	A	Missense
						SNP	342,284	eccA3	T	C	Missense
						Low-frequency variant (27%) ‡	4,327,063	ethA	G	A	-
						Low-frequency variant (25%)	2,837,900	bcp	A	G	-
						SNP	1,813,500	lgt	T	C	Synonymous
						SNP	1,580,949	Rv1404	G	A	Missense
						Low-frequency variant (27%)	1,440,585	cysN	A	G	-
						glpK §	4,139,189	glpK	C	CC	-
						indel	1,707,324	Rv1515c	T	TA	Frameshift
5	5.5	JPN-OP-5-1	SRR26139248	SRR26139259	L2.2. AA1	SNP, back-mutation	3,067,955	-	*T*	*C*	Intergenic
		JPN-OP-5-2	SRR26139247	SRR26139258	L2.2. AA1	SNP	3,774,261	Rv3363c	C	T	Missense
						SNP	2,195,198	mazE5	G	A	Synonymous
						SNP	1,492,131	glgB	T	G	Missense
						SNP	1,030,084	Rv0923c	A	T	Stop-gained
						SNP	762,240	rpoB	G	A	Missense
						SNP	1936	-	A	G	Intergenic
7	3.9	JPN-OP-7-1	SRR26139270	SRR26139255	L2.2. AA2	-	-	-	-	-	-
		JPN-OP-7-2	SRR26139269	SRR26139254	L2.2. AA2	SNP	250,341	Rv0210	G	A	Synonymous
						SNP	615,409	hemL	G	A	Missense
						indel	530,229	groEL2	G	GAG	Frameshift
						indel	1,468,933	Rv1313c	G	GG	Frameshift

* Genomic positions are reported relative to the M. tuberculosis H37Rv reference genome (NC_000962.3).

† REF and ALT denote the nucleotides in the index and secondary isolates, respectively.

‡ In pair 4, three transmitted low-frequency variants that became fixed in the secondary case were excluded from analysis.

§ Indel in glpK was excluded from downstream analyses due to its documented association with in vitro adaptive phenotypes.

Among the remaining six transmission pairs, we identified a total of 28 genomic variant events, affecting 29 distinct sites (Table 2). These included 24 SNPs and three small indels. All three indels resulted in frameshift mutations. In addition, a gene conversion event was observed in pair 3, where a segment of PPE*5* was partially replaced by a sequence from PPE*21*, introducing two tightly linked SNPs. Based on these observations, we estimated an overall mutation event rate of 0.81 (95% CI 0.54–1.18) events per genome per year. As almost all events involved small-scale variants, these findings revealed a landscape of limited genetic divergence. This pattern is consistent with microevolution driven primarily by near-neutral drift under minimal selective pressure.

### 3.3 Recurrent gene conversions and subcluster-specific variants in Bern outbreak

To investigate interpatient microevolution under intensified host-imposed selective pressure, we conducted an analysis on 16 clonally transmitted strains from a 21-year outbreak in Bern, Switzerland (Stritt et al., 2025). For clarity, these long-read sequenced strains were assigned concise identifiers (Table 4). All outbreak strains were classified as lineage L4.4.1.1. As phylogenetic controls, we included the closely related L4.4.1.2 strain N1015 and the geographically distinct New Zealand-derived L4.4.1.1 strain CS1 (Stritt et al., 2025, Mulholland et al., 2022). We identified two primary transmission subclusters and five sporadic cases (Figure 1). Most strains descended from a single ancestral strain, strain 3, which lacked phylogenetically informative markers. Isolated in 1988, its ancestral status is consistent with a proposed central role in initiating the outbreak (Stucki et al., 2015). Strains 10 and strain 16 were excluded from analysis for reasons detailed below.

**Table 4:** Phylogenetic markers and genomic variants identified in the Bern outbreak strains.

Strain	Alias	Marker*	POS_H37Rv	POS_strain3	POS_query	Variant type	Gene/region	REF	ALT	Annotation
Strain5	P008-N1380	Marker1	1951689	1947641	1947641	snp	Rv1725c	C	A	Missense
	(1991)		450161	454457	454457	snp	Rv0373c	G	A	Missense
			503771	508069	508069	snp	lpqL	A	G	Synonymous
			3056018-3056025	3042799-3042806	3042798	Structural-variant	Rv2742c	AATGAGCG	-	Strand slippage, 8bp deletion, frameshift
Strain7	P020-N1386	Marker1	1951689	1947641	1947641	-	Rv1725c	C	A	Missense
	(1992)		1382073	1386981	1386981	snp	corA	C	T	Missense
Strain12	P052-N1429	Marker1	1951689	1947641	1947640	-	Rv1725c	C	A	Missense
	(1995)	Marker2	1176283-1176285	1180131-1180133	1180131-1180132	Complex mutation	Intergenic	-	-	Generating two linked variants upstream of IS6110
			1533546-1533583	1538454-1538491	1538453-1538490	Gene_conversion	ppe19	-	-	Partially replaced by ppe18, introducing 3 variants
			2755176	2743165	2743164	snp	Rv2455c	T	C	Missense
			3346729	3333423	3333421	Homopolymeric tracts	Rv2989	GGGG	GGG	Frameshift
			3844644	3834468	3834466	snp	Intergenic	T	G	-
Strain8	P022-N1431	Marker1	1951689	1947641	1947640	-	Rv1725c	C	A	Missense
	NA	Marker2	1176283-1176285	1180131-1180133	1180131-1180132	-	Intergenic	-	-	Generating two linked variants upstream of IS6110
		Marker3	811425	816556	816556	snp	rplN	A	C	Missense
			653177	657613	657613	grcC1†	grcC1	T	G	Missense
Strain11	P042-N1430	Marker1	1951689	1947641	1947580	-	Rv1725c	C	A	Missense
	(1996)	Marker2	1176283-1176285	1180131-1180133	1180131-1180132	-	Intergenic	-	-	Generating two linked variants upstream of IS6110
		Marker3	811425	816556	816565	-	rplN	A	C	Missense
		.	78548-79556	79584-79592	79593-79601	Structural-variant	Rv0071	-	CCCGGTGGA	Tandem duplication 9bp, conservative_inframe_insertion
			653155	657591	657600	grcC1	grcC1	C	A	Missense
			1218597-1218608	1223764-1223775	1223771	Structural-variant	pe_pgrs22	GTGACGGCGGCG	-	Strand slippage, 12bp deletion, frameshift
			1895535-1895591	1891486-1891542	1891481	Structural-variant	Intergenic			Collapsed tandem repeat, 57bp deletion
			3336745-3336802	3323439-3323496	3323437-3323494	Structural-variant	Intergenic			Acquiring a 1-bp deletion copy of 59-bp repeat unit located upstream of ddlA
			4070697	4064554	4064552	snp	Rv3631	C	T	Missense
Strain1	P001-N1377	Marker4	1572681	1576227	1576226	Homopolymeric tracts	pe-pgrs25	CCCCCCC	CCCCCC	Frameshift
	(1991)	Marker4	1586867	1590408	1590407	snp	Rv1410c	C	T	Synonymous
		Marker4	2075223	2058068	2058067	snp	Rv1830	A	C	Missense
		Marker4	2817672	2804303	2804302	snp	accD1	C	T	Stop_gained
			653165	657601	657601	grcC1	grcC1	C	T	Missense
			3842767-3847692	3832592-3839937	3832591-3839936; 3839937-3847282	Structural-variant		-	-	Tandem duplication, insertion length 7,346 bp
			3847235-3847684	3839480-3839929	3839479-3839928	Gene_conversion	ppe59	-	-	Partially replaced by ppe57, introducing 63 variants
			3928238-3928300	3928399-3928465	3935743	Structural-variant	pe_pgrs53	-	-	A 67-bp deletion removed a region containing multiple types of tandem repeat units.
Strain9	P028-N1362	Marker4	1572681	1576227	1576226	-	pe-pgrs25	C	-	Frameshift
	NA	Marker4	1586867	1590408	1590407	-	Rv1410c	C	T	Synonymous
		Marker4	2075223	2058068	2058067	-	Rv1830	A	C	Missense
		Marker4	2817672	2804303	2804302	-	accD1	C	T	Stop_gained
			653221	657657	657657	grcC1	grcC1	T	A	Missense
			4002338	3995973	3995972	snp	fadD3	G	A	Synonymous
Strain15	P073-N1394	Marker4	1572681	1576227	1576226	-	pe-pgrs25	C	-	Frameshift
	(1998)	Marker4	1586867	1590408	1590406	-	Rv1410c	C	T	Synonymous
		Marker4	2075223	2058068	2058066	-	Rv1830	A	C	Missense
		Marker4	2817672	2804303	2804301	-	accD1	C	T	Stop gained
			467500	471797	471796	Homopolymeric tracts	ppe9	GGGG	GGG	Frameshift, a back mutation
			3123103-3123247	3107797-3107941	3107793	Structural-variant	Intergenic	-	-	A 145 bp deletion from tandem repetitive array within 4,392 bp MTBC-specific island; deleted fragment shares 77–83% identity with 145 bp flanking loci.
			3346750	3333444	3333296	snp	Rv2989	T	C	Missense
			3805209	3794802	3794654	snp	lpqD	A	C	Missense
Strain2	P003-N1374		653221	657657	657656	grcC1	grcC1	T	C	Missense
	(1991)		1339399-1339905	1344306-1344812	1344305-1344811	Gene_conversion	ppe18	-	-	Ppe18 of ancestral strain 3 partially replaced by ppe19 with 14 variants‡; in Strain 2, a further replacement by ppe19 fully overlapping prior region and introducing 6 variants.
			325644	327103	327102	indel	fadD2	G	-	Missense
			523068	527366	527365	snp	Rv0435c	G	A	Missense
			852634	857765	857764	snp	phoR	G	C	Missense
			970077	973910	973909	snp	pe_pgrs15	G	A	Synonymous
			1955274	1951226	1951224	Homopolymeric tracts	Rv1729c	CCCCCC	CCCCC	Frameshift
Strain4	P007-N1108		1533208	1538116	1538116	snp	ppe19	C	G	Synonymous
	(2005)		2390561	2378523	2378523	snp	Rv2129c	C	T	Stop_gained
			2401673	2389635	2389635	snp	Rv2141c	T	C	Missense
			3408959	3395657	3395657	snp	nrdF2	G	A	Synonymous
			3968952	3962587	3962587	snp	Intergenic	A	T	-
			4115367	4109223	4109223	snp	nth	C	A	Missense
			4139189	4133046	4133047	glpK	glpK	-	C	Synonymous
Strain6	P010-N1385		653165	657601	657601	grcC1	grcC1	C	T	Missense
	(1991)		1899953	1895904	1895903	indel	Intergenic	T	-	-
			2639367	2623392	2623391	snp	Intergenic	A	C	-
Strain10	P034-N1426		652826	657262	657262	grcC1	grcC1	C	T	Missense
	(1995)		830636	835767	835767	snp	Intergenic	C	T	-
			867288	872417	872417	snp	ggtA	G	C	Synonymous
			1341624	1346531	1346531	snp	IS1081	G	T	Missense
			2257871	2242215	2242215	snp	Rv2008c	G	A	Synonymous
			3177505	3163566	3163566	snp	Intergenic	G	C	-
			3232755-3232866	3219206	3219207-3219318	Structural-variant	Intergenic	-	-	Acquiring two tandem 56-bp repeats in intergenetic region.
			772908	777877	777877	Ancestral state	fadE8	G	C	Missense
			1342946	1347853	1347853	Ancestral state	Rv1200	G	A	Missense
			2043368	2026334	2026334	Ancestral state	Integenic	G	A	Missense
			3063788	3050569	3050569	Ancestral state	Rv2751	A	C	Synonymous
			3121273	3111012	3111012	Ancestral state	IS6110	C	A	Missense
			3522352	3508827	3508939	Ancestral state	nuoL	C	G	Missense
			3883915	3876159	3876271	Ancestral state	Rv3466	A	G	Missense
Strain13	P059-N1392		653223	657659	657659	grcC1	grcC1	C	G	Missense
	(2002)		3594375-3594455	3578073-3578184	3578072	Structural-variant	Intergenic	-	-	Loss of two tandem 56-bp units in intergenic region
			1636757-1637264	1640544-1641039	1640544-1641038	Gene_conversion	pe_pgrs28	-	-	Two conversions transferring two segments from pe-pgrs27, introduced 32 variants.
			3303903	3290243	3290243	snp	Rv2951c	G	A	Missense
strain14	P066-N1411		653221	657657	657657	grcC1	grcC1	T	C	Missense
	(2003)		300141	301599	301599	snp	Rv0248c	G	A	Synonymous
			1096398	1100243	1100243	snp	pe_pgrs18	C	T	Synonymous
			3028569	3015356	3015356	snp	Rv2715	G	A	Missense
			3661899	3645625	3645625	snp	birA	G	A	Synonymous

* Markers are indicated only upon first appearance in the phylogenetic tree.

† Variants in *grcC1* and *glpK* were excluded from downstream analyses due to their known association with in vitro adaptive phenotypes.

‡ An early, fixed *ppe18/ppe19* gene conversion originated in the common ancestor of the outbreak clade, was fixed in all 15 long-read sequenced outbreak strains (L4.4.1.1), and is absent in related control strains (N10159, L4.4.1.2; CS1, L4.4.1.1), supporting its association with transmission advantage.

**Figure 1:**
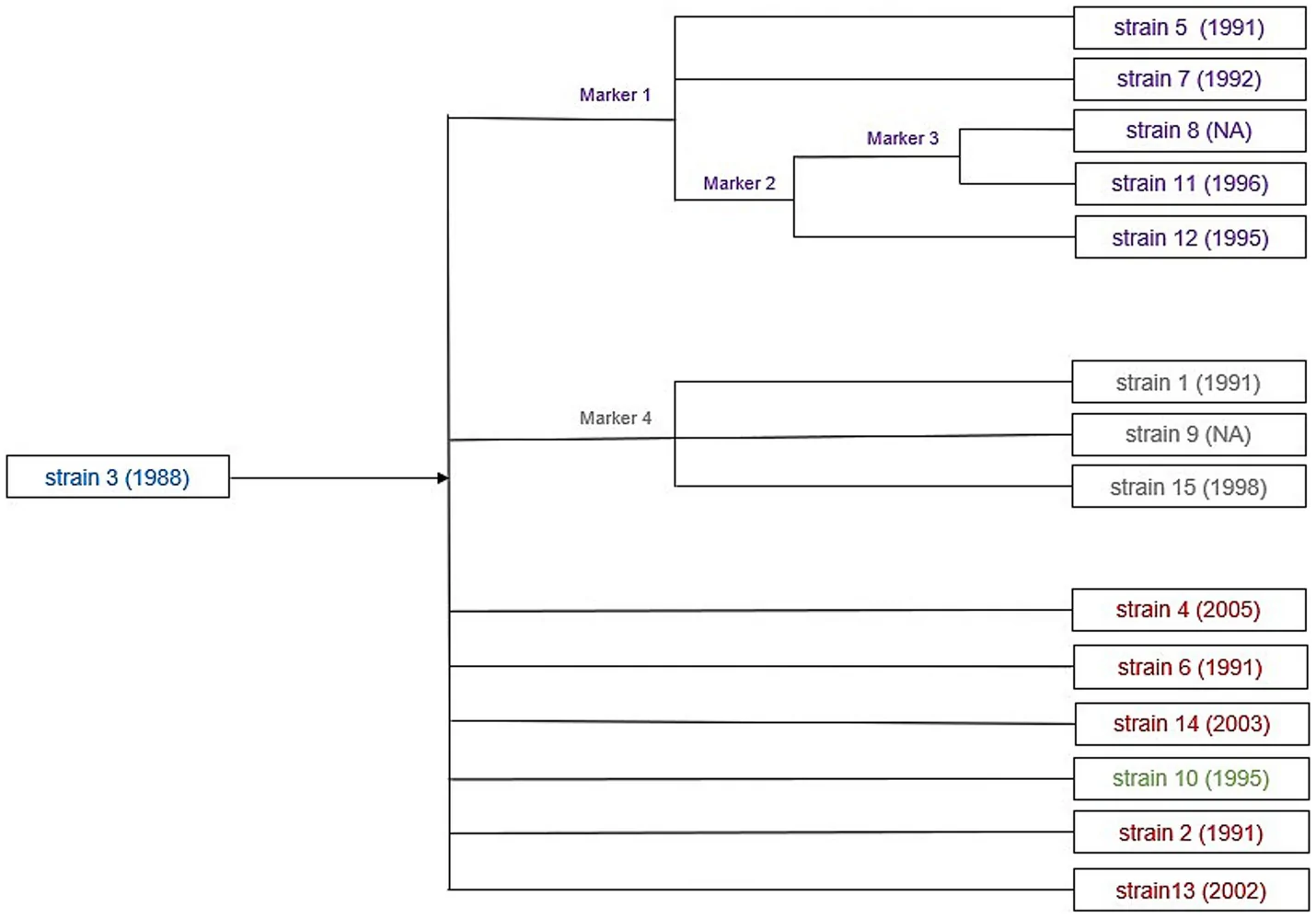
Long-read genomic phylogeny and signature variants of the Bern outbreak Schematic representation of phylogenetic relationships among 15 *M. tuberculosis* strains from the Bern outbreak. Branch lengths are illustrative. Isolates are labelled with year of isolation. Strain 3 is inferred as the ancestral node. Coloured branches denote phylogenetic subclusters: purple, subcluster 1; grey, subcluster 2; red, sporadic isolates. Strain 10 (light green) derives from a paralogous lineage, and strain 16 was omitted due to evidence of cross-contamination. Marker 2 corresponds to a complex mutation generating two tightly linked variants. Marker 4 represents a set of four variants.

Subcluster 1 was defined by the shared SNP NC_000962.3: 1951689 C > A (Table 4). Subcluster 1–1 comprised strains 5 and 7. Strain 5 harbored an 8-bp deletion (AGCGAATG) within a region flanked by direct AGCG repeats in the ancestral genome, a pattern characteristic of replication slippage. Subcluster 1–2 descended from subcluster 1–1 and consisted of strain 12, which had experienced two independent recombination events. One event generated a complex mutation yielding two tightly linked intergenic variants upstream of an *IS6110* element, serving as a robust phylogenetic marker for this subcluster. The other involved a gene conversion event in which a 38-bp segment of PPE19 was replaced by a homologous tract from PPE18, introducing three variant sites. Subcluster 1–3, which evolved from subcluster 1–2 and included strains 8 and 11, was distinguished by an additional marker (NC_000962.3: 811425 A > C). Notably, the intergenic region upstream of *ddlA* in ancestral strain 3 contained six copies of a 59-bp repeat unit, interleaved with four derived copies harboring an identical SNP and one copy bearing a single-bp deletion. In contrast, strain 11 carried an extra copy of the 1-bp-deletion variant. These complex repetitive architectures, with minimal sequence variations, highlight the substantial challenges they pose for automated genome annotation.

Subcluster 2 comprised strains 1, 9, and 15, all sharing an identical set of four variant markers (Table 4). Strain 1 harbored a 7,346 bp duplication, within which a segment of PPE*59* was replaced via gene conversion by a homologous region fromPPE*57*. This event introduced 63 variants, as previously reported (Stritt et al., 2025). Additionally, within the PE_PGRS53 gene of strain 1, the deletion of multiple tandem repeat units resulted in a cumulative loss of 67 bp. In contrast, strain 9 exhibited remarkable genomic stasis, harboring only a single synonymous SNP. In strain 15, a 145 bp fragment was deleted from a tandem array of interspersed repetitive elements located within a 4,392 bp MTBC-specific genomic island. This fragment shares high sequence similarity (77–83% identity) with multiple flanking loci, each approximately 145 bp in length. Long-read sequencing initially suggested that strain 16 was identical to strain 15. However, corresponding short-read data revealed that strain 16 possessed the two earliest phylogenetic markers specific to subcluster 1 but lacked the variants defining subcluster 2. This discrepancy strongly indicates that strain 16 actually belongs to subcluster 1 and that its long-read dataset was likely contaminated.

Five sporadic isolates were defined by distinct lineage-specific variants (Table 4). A key finding was recurrent gene conversion at the PPE18/PPE19 locus. In strain 2, a segment of PPE18 was replaced by a homologous tract from PPE19, introducing six variant sites. This event appeared to extend an earlier gene conversion that originated in ancestral strain 3, where a smaller PPE19-derived fragment had introduced 14 variant sites into PPE18. The new conversion in strain 2 fully encompassed this initial fragment. In contrast, the PPE18 gene remained intact in the phylogenetically proximate control strains CS1 and N1015, underscoring that these sequential gene conversion events represent a distinctive evolutionary signature of the Bern outbreak lineage. Recurrent homologous recombination was also observed in strain 13. As reported previously, this involved two adjacent segments of PE-PGRS28 (191 bp and 32 bp) being replaced by homologous sequences from PE-PGRS27, introducing 32 variant sites (Stritt et al., 2025). This pattern mirrors the recurrent PPE18/PPE19 conversions and likely resulted from two independent gene conversion events.

Despite being isolated 15 and 17 years apart, strains 14 and 4 exhibited exceptional genomic stability, accumulating only four and six SNPs respectively, most of which were synonymous. In addition to the PPE18/PPE19 conversions observed in other strains, we also identified a synonymous variant in PPE19 in strain 4. Strain 6 exhibited similarly limited divergence. Strain 10, isolated in 1995, diverged from strain 3 through the acquisition of two additional copies of a 56-bp tandem repeat and the accumulation of 12 SNPs. Notably, at seven of these positions, strain 10 represent the ancestral state. As discussed later, strain 10 originated from a lineage paralogous to that of strain 3 and was therefore excluded from the core analysis.

Following phylogenetic reconstruction, we identified 50 mutational events across 13 isolates descending from ancestral strain 3. Among the 11 strains with available isolation dates, the overall mutation event rate was 0.55 (95% CI 0.42–0.74) per genome per year— a value comparable to that observed under low-pressure settings. Variants were further categorized by molecular origin to assess their potential functional impact. The resulting spectrum was dominated by 29 SNPs, followed by large-scale variants including gene conversions and structural variants, along with four homopolymeric tracts (Table 2). To assess the impact of gene conversions and enable comparison with conventional analytical frameworks based on individual variant sites, we also quantified the number of affected genetic sites per event and presented these values in a separate column in Table 2. Notably, the five newly emerging gene conversion events collectively introduced a total of 104 variant sites.

To test whether the types of variants differed between latent infections and the clonal outbreak, and given the limited sample size, we stratified the variants into two categories: small-scale variants (SNPs and indels), which are reliably detectable by short-read sequencing; and more complex variants, including larger-scale gene conversions, structural variants, and homopolymeric tracts, which are best resolved by long-read sequencing. We then compared the distributions of these two categories between the two epidemiological settings.

Mann–Whitney U tests were performed to compare the annual rates of different categories of variants between latent pairs (*n* = 6) and outbreak strains (*n* = 11). No significant difference was observed for small-scale variants (U = 17, *p* = 0.116). In contrast, the outbreak group exhibited a significantly higher accumulation rate of complex variants (U = 14, *p* = 0.04). The effect was particularly amplified when considering large-scale gene conversions and structural variants, which introduce multiple linked changes en bloc. Although the small sample size warrants caution in extrapolation, these results indicate that the outbreak is characterized by a specific enrichment of larger-scale variants. Moreover, these observations suggest that the pathogen can shift its evolutionary mode, from small-scale mutations to large-scale rearrangements, when facing intense host-imposed selective pressure.

In the original long-read study, singletons were defined as variants observed in only one of the sequenced isolates (Stritt et al., 2025). Consistent with strong purifying selection, the majority of mutation events in our analysis were transient singletons (86%, 43/50)—including all newly emerging gene conversions and structural variants. The early gene conversion event at the PPE18/PPE19 locus was excluded from this count, as it originated in ancestral strain 3 and became fixed across its entire descendant lineage, representing an ancestral, lineage-defining mutation rather than a newly arising singleton. This predominantly singleton pattern was further confirmed across the full cohort of 74 strains using short-read data (detailed below), demonstrating that only a small set of variants survived selection and served as phylogenetically informative, subcluster-specific markers.

### 3.4 Assessing the phylogenetic resolution of subcluster-specific SNPs using short-read data

To evaluate the phylogenetic resolution conferred by subcluster-specific SNPs initially identified through long-read sequencing, we reconstructed a maximum-likelihood phylogeny using short-read data from 74 strains. Thirteen of these strains had also undergone long-read sequencing and served as internal controls. The phylogenetic structure was robust. A maximum-likelihood tree built from 3,255 high-quality SNP sites derived from short-read data delineated two major subclusters and several sporadic cases. This topology was fully consistent with that inferred from long-read data (Figure 2).

**Figure 2:**
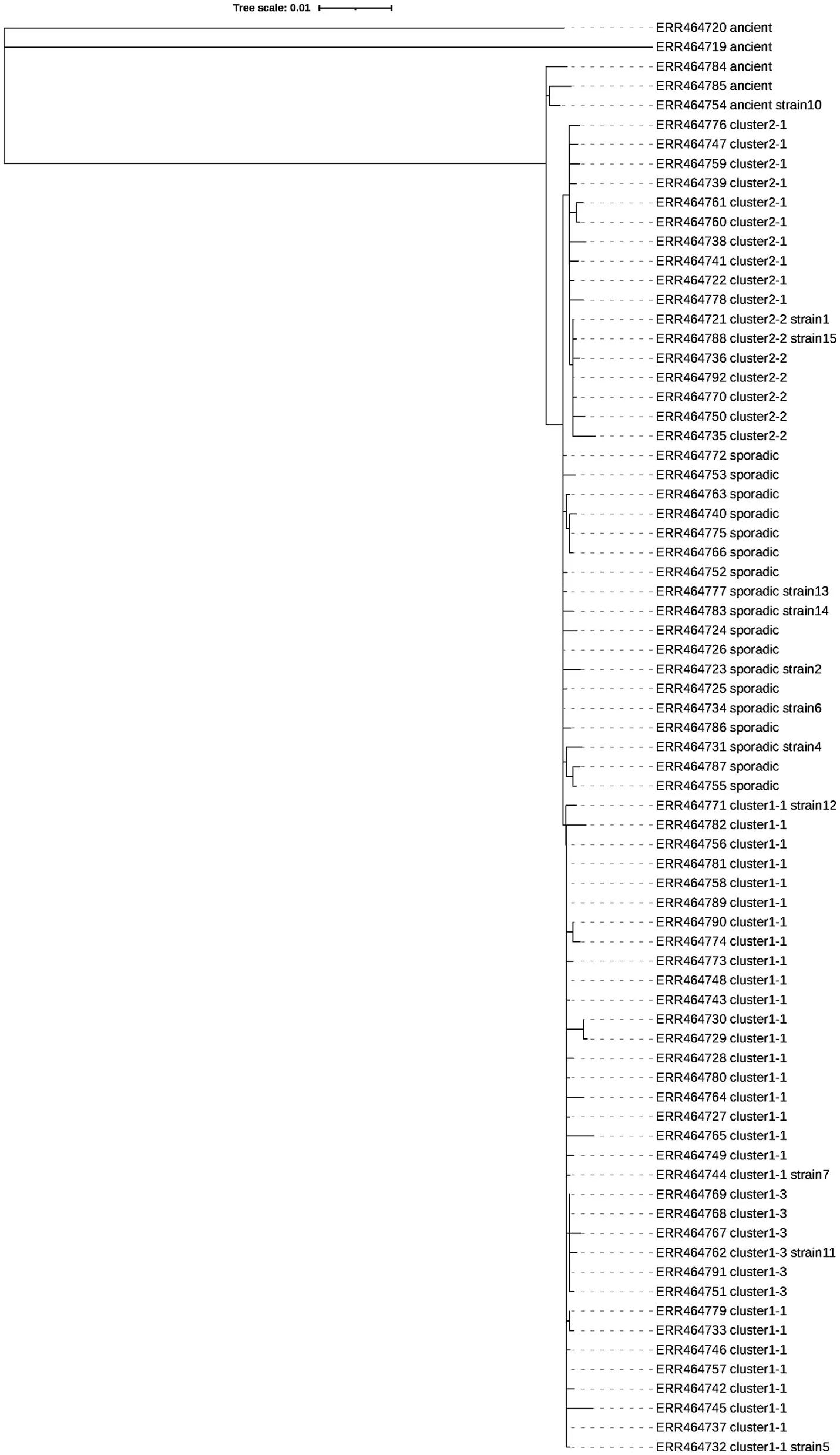
Phylogenetic reconstruction of the Bern outbreak based on short-read sequencing. The tree was built from 3,255 high-quality SNP sites across 74 M. tuberculosis strains. Thirteen strains with long-read data served as internal controls. The topology is consistent with that derived from long-read sequencing.

Subcluster 1 comprised 34 strains, representing nearly half of the total cohort (Figure 2; Supplementary Table S3). However, the phylogenetic marker defining subcluster 1–2, consisting of two tightly linked variants located upstream of an *IS6110* insertion, could not be reliably resolved using short-read data. As a result, subclusters 1–2 and 1–3 were collapsed into a single branch in the short-read phylogeny. Consequently, whereas strain 12 was inferred as the direct ancestor of strain 11 in the long-read tree, it appeared only as its sister lineage in the short-read reconstruction. Subcluster 2 included 17 strains, accounting for approximately one-quarter of the collection. The four variants defining this group in the long-read data were not uniformly shared but instead accumulated sequentially across its sublineages. Specifically, subcluster 2–1 harbored two core SNPs, one missense (NC_000962.3: 2075223 A > C) and one synonymous (NC_000962.3: 1586867C > T), while subcluster 2–2 acquired an additional stop-gain mutation in the accD1 gene (Supplementary Table S3). Another sublineage-defining variant, a slipped-strand mutation causing a frameshift in PE-PGRS25, was excluded from the analysis due to its unreliable detection in short-read data.

A notable topological discrepancy involved the placement of ancestral strain 3, which clustered among sporadic isolates in the short-read phylogeny. This misplacement arose because the phylogeny construction pipeline treated both sporadic isolates and the inferred ancestral state identically, as neither carried phylogenetically informative SNPs. Beyond the major subclusters, the phylogeny also resolved two minor sporadic groups, comprising four and two isolates, respectively. Within subclusters 1 and 2, four additional daughter clusters, each containing two strains, were further delineated.

Comparative analysis of long-read data revealed 12 SNPs differentiating strain 10 from strain 3. At seven of these loci, strain 10 retained ancestral alleles, whereas all other long-read-sequenced outbreak strains shared the derived genotype of strain 3. We therefore examined the distribution of these seven ancestral SNPs across all 74 short-read sequenced strains. Two SNPs fell within RLC regions and were excluded. Among the remaining five SNPs, only four strains retained the ancestral alleles observed in strain 10, all others matched strain 3. Phylogenetically, these four strains, together with strain 10, formed a distinct sporadic subcluster that branched basally to the major lineages descending from strain 3. This topology suggests that these five isolates originated from an early-diverging sister lineage to the main outbreak clade.

To assess the generalizability of singletons, we expanded our analysis to the full cohort of 74 outbreak strains, tracking the population-wide distribution of the 34 SNPs initially identified through long-read sequencing. Of these, three sites were consistently annotated as LowCov and were excluded (Supplementary Table S3). At two additional sites, no variant alleles were detected in any of the 74 strains, suggesting that these mutations likely arose artifactually during strain storage or revival. Moreover, two SNPs (NC_000962.3: 450161 G > A and 503,771 A > G) called in the long-read assembly of strain 5 were absent in its corresponding short-read dataset (ERR464732) but were uniquely present in another sample (ERR464749). This discrepancy provides strong evidence of cross-sample contamination while still supporting the singleton status of these variants. Excluding these anomalies, only one non-singleton SNP (NC_000962.3: 867288 G > C) was identified in two isolates (strain 10 and ERR464785); all remaining SNPs were confirmed as true singletons, each detected in exactly one isolate across the entire 74-strain cohort.

## 4 Discussion

In *M. tuberculosis*, conventional estimates of evolutionary dynamics have overwhelmingly relied on the accumulation of SNPs, which are typically treated as discrete and independent mutational events (Ley et al., 2019; Vargas et al., 2021). This SNP-centric paradigm has been widely adopted, largely because of its analytical tractability and ready applicability to phylogenetic and selection scan analyses (Stritt et al., 2025). Another reason is that short-read sequencing, the dominant platform in tuberculosis genomics, has limited ability to accurately resolve repetitive or GC-rich regions. This is particularly true for the PE/PPE gene families, leaving a substantial fraction of the genome inaccessible (Marin et al., 2022). Although long-read sequencing now provides access to these previously inaccessible regions, significant computational challenges remain in the reliable detection and interpretation of complex variants (Marin et al., 2025). As a pragmatic compromise, current evolutionary models often conflate mutations arising from fundamentally distinct molecular mechanisms into a single, undifferentiated count of variant sites. However, mutations originating from different processes frequently produce divergent genomic outcomes. For instance, a single gene conversion event can replace an extended homologous segment, simultaneously introducing multiple linked nucleotide changes (Stritt et al., 2025). Similarly, structural variants can dramatically reshape large chromosomal regions (Canalda-Baltrons et al., 2025; Modlin et al., 2026).

Our analysis suggests that the numbers of mutational events were comparable across distinct epidemiological settings. This refined view challenges the conventional assumption that outbreaks are driven by elevated mutation rates. Instead, it raises the possibility of a possible shift in the types of mutations that accumulate: under low host-imposed pressure, evolution is largely characterized by small-scale changes, whereas under intense selection, larger-scale alterations such as gene conversions and structural variants may become more prominent. This distinction is often obscured when variants are aggregated as individual nucleotide sites in conventional SNP-based analyses. Such conflation can compromise molecular clock calibrations and mask genuine adaptive signals. Collectively, these findings suggest that accounting for the molecular mechanisms underlying different mutation types may offer additional insight beyond conventional site-based counting approaches.

Within-host mutation rates in *M. tuberculosis* strains vary substantially, ranging from 0.3 to 7.0 SNPs per genome per year (Ley et al., 2019). A large-scale study also reported a SNP accumulation rate of 0.56 SNPs per genome per year (Vargas et al., 2021). Numerous within-host studies have described that *M. tuberculosis* isolates follow a clear trajectory from susceptibility to multidrug resistance through sequentially acquired drug-related mutations (Saunders et al., 2011; Eldholm et al., 2014; Bloemberg et al., 2015; Sonnenkalb et al., 2021; Yokobori et al., 2022). In contrast, another study found that the *M. tuberculosis* genome remained highly stable in a poorly adherent, HIV-positive host, indicating an absence of host and antibiotic selective pressures (Perez-Lago et al., 2015). This aligns with the relatively low within-host mutation rate of 0.36 events per genome per year observed in our study. SNP stability in *M. tuberculosis* has been linked to the intensity of antibiotic pressure (Trauner et al., 2017). Drug resistance-conferring loci also evolved at a higher within-host SNP rate (seven SNPs per genome per year) than other genomic regions (1.1 SNPs per genome per year; Ley et al., 2019). All these observations indicate that the mutation rate within the same host is shaped by the pressures imposed by both the host and antibiotics.

We also observed a mutation rate of 0.81 events per genome per year in latent household isolates. A study involving 24 pairs of latent household strains described a similar average mutation rate of 0.85 (95% CI: 0.62–1.09) SNPs per genome per year (Colangeli et al., 2020). However, it is worth noting that the mutation accumulation rates reported in this study should be compared with those from previous studies with caution, as our estimates capture the complete landscape of genetic variation – including the PE/PPE family, which is typically excluded from conventional short-read studies. Furthermore, if large-scale gene conversions and structural variants occurred and were counted as individual variant sites, the mutation rates would increase dramatically.

Our analysis proposed that the microevolutionary pattern in outbreak setting was characterized by the introduction of large-scale variants. Gene conversions and structural variants introduced multiple genetic changes en bloc, reflecting a coordinated adaptive response to intense host-imposed diversifying pressures. Notably, the two strains exhibiting gene conversions that introduced the largest number of variant regions also carried structural variants, and this co-occurrence suggests that both types of alterations arose under similarly strong host-mediated selection. Such diversifying selection was further evidenced by recurrent gene conversion events within the immunologically critical PE/PPE gene families. Specifically, we identified two novel conversion events and a synonymous SNP at the PPE18/PPE19 locus among the outbreak strains. Moreover, an earlier conversion event, wherein a short PPE19-derived fragment was integrated into PPE18, originated in the ancestral strain 3 and became fixed across its entire descendant lineage. The absence of this allele in phylogenetically related control strains further supports its selective advantage. Collectively, the recurrence of conversions at this locus indicates that it has been a focal target of local diversifying selection.

PPE18 and PPE19 are highly homologous secreted proteins that exhibit functional redundancy (De Voss et al., 2025). The selection of PPE18 as a vaccine component was justified by its ability to elicit a Th1-biased immune response (Von Eschen et al., 2009). However, PPE18 also interacts with the LRR 11–15 domain of TLR2, leading to the induction of IL-10 and a shift toward Th2-type immunity, which suppresses the Th1-type response required for host defense against *M. tuberculosis* (Manikoth et al., 2025). Similarly, murine infection models have demonstrated the role of PPE19 in promoting *M. tuberculosis* entry into host macrophages (De Voss et al., 2025). High sequence variability of PPE18 has also been reported, consistent with the recurrent gene conversion events observed in this study, indicating intensive diversifying selection (Homolka et al., 2016; Garcia-Marin et al., 2026). It is possible that the early gene conversion between PPE18 and PPE19 could produce a chimeric protein, which may augment the virulence of this pathogen through specific mechanisms. Nevertheless, this hypothesis calls for rigorous functional validation. These findings align with broader patterns of pathogen adaptation. For instance, divergent evolution of the esxH gene in Lineage 1 strains from South and Southeast Asia reflects positive selection favoring local adaptation to sympatric host populations (Menardo et al., 2021). Similarly, loss-of-function mutations in sseA among Tibetan Plateau isolates facilitate adaptation to high-altitude, low-oxidative-stress environments (Liu et al., 2021).

Concurrently, the majority of genetic variants identified in the Bern outbreak strains were transient singletons, including all newly emerging gene conversion and structural variants. This prevalence of singletons is a hallmark of purifying selection. The *Mycobacterium tuberculosis complex* (MTBC) is renowned for its exceptionally low genetic diversity, indicative of stringent genome-wide evolutionary constraints (Ley et al., 2019). This constraint has been well documented across both between-host and within-host scales of microevolution (Namouchi et al., 2012, Liu et al., 2020). A quintessential manifestation is the well-established fitness cost associated with many antimicrobial resistance mutations (Castro et al., 2021). Ecologically, MTBC lineages are typically maintained within specific, sympatric human populations, reflecting a long-standing history of host–pathogen coevolution. This stable association, however, can be disrupted in contexts such as HIV co-infection (Gagneux et al., 2006; Fenner et al., 2013).

Accordingly, the “evolutionary optimum” hypothesis posits that MTBC strains have already attained a high degree of host adaptation (Stritt and Gagneux, 2023). As a result, evolutionary change is now largely driven by neutral or deleterious variants, allowing purifying selection to dominate. This effect is further amplified by the extreme clonality and strict genomic linkage inherent to MTBC, resulting from the absence of horizontal gene transfer. Such a clonal population structure promotes background selection, efficiently purgeing linked deleterious or hitchhiking mutations (Stritt and Gagneux, 2023). Together, background and purifying selection act synergistically to enforce the remarkable genomic stability characteristic of MTBC. In contrast, when *M. tuberculosis* strains are exposed to drastic environmental alterations—including heightened immune pressures during inter-host transmission in outbreaks—they adapt to strong diversifying selection primarily through large-scale variants. This shift is accompanied by a concomitant enhancement of purifying selection, which eliminates an elevated proportion of SNPs, many of which represent hitchhiking mutations or products of genetic drift. Similar discordance between structural variants and SNP diversity patterns has also been observed in other species. For instance, the invasive Australian population of the European starling exhibited lower SNP diversity but higher structural variants diversity compared to the native UK population (Stuart et al., 2023).

In this study, only the ancestral, lineage-fixed gene conversion at the PPE18/PPE19 locus and a small set of SNPs survived purifying selection. Several of these SNPs, some located within PE/PPE family-associated genes such as PE-PGRS25 and accD1, became fixed in some outbreak sublineages. These observations suggest that the PPE18/PPE19 conversion played a pivotal role in driving the outbreak’s expansion, while the surviving SNPs may provide additional functional advantages. A central conundrum in *M. tuberculosis* biology is how a remarkably successful global pathogen can maintain an exceptionally stable and clonal genome (Hershberg et al., 2008). Our findings suggest a nuanced evolutionary dynamic during outbreaks: (i) Episodic bursts of adaptive diversification, driven by heterogeneous selective pressures, generate a transient pool of genetic variants. (ii) This pool is then rigorously filtered by the strong purifying and background selection, thereby preserving extremely low genomic diversity. (iii) However, a select few beneficial variants evade this filtering, confer substantial functional advantages, and ultimately give rise to epidemiologically dominant sublineages. Our observations suggest that this dynamic equilibrium between diversifying innovation and stringent purifying control appears to shape the adaptive landscape of *M. tuberculosis* in the context of clonal transmission.

## 5 Limitations

This study has several limitations. First, the limited availability of long-read sequencing data constrains the sample size involved. Second, as this is a preliminary investigation, the findings necessitate cautious interpretation. Further validation through more stringent statistical analyses and expanded data sets is warranted.

## Data Availability

The datasets presented in this study can be found in online repositories. The raw sequencing data for the three novel within-host strains are deposited in the Genome Sequence Archive under accession CRA032293 (https://ngdc.cncb.ac.cn/gsa/). Publicly available data for the household transmission cohort were obtained from NCBI BioProject accessions PRJNA1019874 and PRJNA1087460. Data for the Bern outbreak cohort were sourced from NCBI BioProject accessions PRJEB73759 (PacBio long-read) and PRJEB5925 (Illumina short-read).

## Footnotes

1
https://itol.embl.de/
