# Supplementary material for: Recurrent gene conversions and subcluster-specific variants are associated with Mycobacterium tuberculosis adaptation to intensive host pressure: a comparative genomic study
Source: Front Microbiol. 2026 Oct 7;17:1950486. doi: 10.3389/fmicb.2026.1950486 (PMC13683665; doi:10.3389/fmicb.2026.1950486)
Supplement: Supplementary file 1 [file Table_4.DOCX]

Supplementary File S1: Calculation of mutation event rates

Overview

We used long-read derived genomic data to estimate the rate of mutational events across different epidemiological settings.

Definition of mutational events

A mutational event was defined as any independently occurring genetic alteration, including SNPs, small indels, homopolymeric tract variations, gene conversions, and structural variants. Each event was counted once regardless of the number of nucleotide sites affected.

Sample exclusion and variant filtering criteria

1) All long-read assemblies were validated by even short-read coverage, and assemblies with non-uniform coverage suggestive of misassembly were excluded. 2) Variants that were present as low-frequency polymorphisms in the index case and subsequently fixed in the secondary case were excluded, as they were not considered de novo mutations acquired during the secondary infection.

Cohort-specific calculations

For each isolate or transmission pair, the rate was calculated as the number of events divided by the time interval between the available isolation dates, expressed as events per genome per year. The rate of mutational event was calculated between strains representing a linear descendant relationship, while the strain originating from a paralogous sublineage was excluded.

- Within-host pair

The mutational event rate was calculated between strain 191350 and 227375. Strain 211397 was excluded, as it likely originated from a paralogous sublineage within the same host.

- Latent household transmission pairs

The rate was calculated for each of the six included pairs, and pairwise rates were averaged to obtain an overall estimate. 2) Pair 6 was excluded because the long-read assembly of isolate JPN-OP-6-1 contained a contig with substantially reduced short-read coverage (97.85%), indicating possible incompleteness. 3) Three SNPs in pair 4 were present at low frequencies (25–27%) in the index case and later became fixed in the secondary case. Because these variants pre-existed in the index case rather than arising de novo during the secondary infection, they were excluded from downstream analyses.

- Bern outbreak strains

The rate was calculated for each strain relative to the ancestral strain 3, and the overall rate was estimated as the mean of these per-strain rates. Strain 10 was excluded as it likely originated from a paralogous lineage, and strains 8 and 9 were excluded due to unavailable isolation dates.

Statistical analysis

1) To compare the types of genetic variants accumulated under different epidemiological settings, we stratified all identified mutational events into two categories: (i) small-scale variants (SNPs and indels), which are reliably detectable by short-read sequencing; and (ii) complex variants (gene conversions, structural variants, and homopolymeric tract variations), which are best resolved by long-read sequencing. 2) Mann-Whitney U tests were used to compare the annual rates of small-scale and complex variants separately. The comparisons were made between latent household transmission pairs and Bern outbreak strains. All statistical analyses were performed using SPSS (version 22), and a two-tailed p-value < 0.05 was considered statistically significant.

Output data

Summary output data are provided in Table 2 of the main text.
